# Axon-Seq Decodes the Motor Axon Transcriptome and Its Modulation in Response to ALS

**DOI:** 10.1016/j.stemcr.2018.11.005

**Published:** 2018-12-11

**Authors:** Jik Nijssen, Julio Aguila, Rein Hoogstraaten, Nigel Kee, Eva Hedlund

**Affiliations:** 1 Department of Neuroscience, Karolinska Institutet, Stockholm 171 77, Sweden; 2 Department of Translational Neuroscience, Brain Center Rudolf Magnus, UMC Utrecht, Utrecht 3984 CG, Netherlands

**Keywords:** RNA sequencing, microfluidic devices, amyotrophic lateral sclerosis, motor neurons, stem cells, transcription factors

## Abstract

Spinal motor axons traverse large distances to innervate target muscles, thus requiring local control of cellular events for proper functioning. To interrogate axon-specific processes we developed Axon-seq, a refined method incorporating microfluidics, RNA sequencing (RNA-seq), and bioinformatic quality control. We show that the axonal transcriptome is distinct from that of somas and contains fewer genes. We identified 3,500–5,000 transcripts in mouse and human stem cell-derived spinal motor axons, most of which are required for oxidative energy production and ribogenesis. Axons contained transcription factor mRNAs, e.g., *Ybx1*, with implications for local functions. As motor axons degenerate in amyotrophic lateral sclerosis (ALS), we investigated their response to the *SOD1*^*G93A*^ mutation, identifying 121 ALS-dysregulated transcripts. Several of these are implicated in axonal function, including *Nrp1*, *Dbn1*, and *Nek1*, a known ALS-causing gene. In conclusion, Axon-seq provides an improved method for RNA-seq of axons, increasing our understanding of peripheral axon biology and identifying therapeutic targets in motor neuron disease.

## Highlights

• Axon-seq improves purity, sensitivity, robustness, and cost of RNA-seq of axons
• Human and mouse motor axons contain mRNAs for energy production and translation
• Motor axons contain transcription factor mRNAs, e.g., Ybx1, implying local functions
• Axon-seq reveals axonal dysregulation of mRNAs in ALS, including Nrp1, Dbn1, and Nek1

## Graphical Abstract

**Figure:**
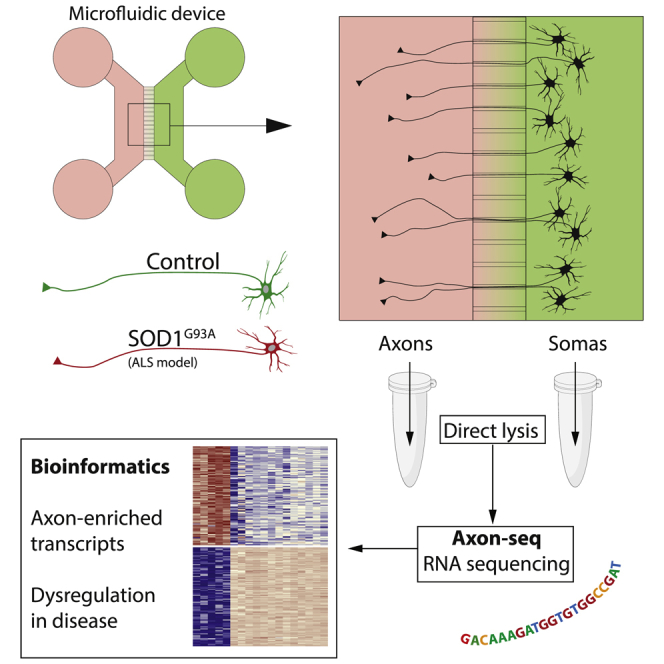

## Teaser

In this article, Nijssen, Aguila, and colleagues report an improved method for RNA sequencing of axons, Axon-seq, that incorporates microfluidics and stringent bioinformatic quality control. The authors show that the axonal transcriptome is smaller than and distinct from that of somas and contains genes required for oxidative energy production and ribogenesis as well as a unique set of transcription factors. Axon-seq reveals that the ALS-causing SOD1^G93A^ mutation dysregulates transcripts with axonal functions.

## Introduction

Spinal motor neurons (MNs) are highly polarized cells. Their somas and dendrites are located in the spinal cord, while their axons traverse the body and connect to muscle fibers. The large distance between the MN soma and its synapse implies that the distal axon must contain a microenvironment able to independently respond to internal and external triggers. Vesicles containing proteins and RNAs travel slowly, at 0.1–10 mm per day (Lasek et al., 1984). Thus, protein transport alone does not suffice to sustain the dynamics of the axon and synapse. Local synaptic translation is important for temporal control of protein synthesis and synaptic plasticity (Holt and Schuman, 2013).

Motor axons and their specialized synapses with muscle, termed neuromuscular junctions (NMJs), are primary targets in the lethal disease amyotrophic lateral sclerosis (ALS). MNs undergo progressive degeneration in ALS, resulting in denervation of muscle, paralysis, and ultimately death at 3–5 years post-diagnosis (Swinnen and Robberecht, 2014). Notably, MNs follow a distinct “dying-back” pattern of degeneration in ALS. Muscle denervation and axonal retraction occur before MN somas in the spinal cord are lost, implying that the NMJ is a highly vulnerable entity of the MN (Comley et al., 2016, Fischer et al., 2004). Mutations in several genes, including *SOD1*, *C9orf72*, *TARDBP*, and *FUS*, cause ALS (DeJesus-Hernandez et al., 2011, Kwiatkowski et al., 2009, Rosen et al., 1993, Sreedharan et al., 2008, Vance et al., 2009). However, expression of these genes is not MN restricted. Why and how MNs are selectively affected in ALS is unclear. Studying the RNA composition of the distal MN axon may illuminate mechanisms driving the specific dying-back degeneration observed in ALS.

Previous efforts to isolate axons *in vitro* have used well insets, Campenot chambers, or microfluidic devices (Boyden, 1962, Campenot, 1977, Taylor et al., 2005). While these can separate axons from somas, residual cross-contamination between compartments can still occur. Thus, axonal fractions can be contaminated with other cellular components or non-neuronal cells. In this way, RNA-sequencing efforts aimed at isolating the axonal transcriptome (Briese et al., 2016, Minis et al., 2014, Rotem et al., 2017, Saal et al., 2014) can be easily undermined if the purity of the axonal fractions is not carefully examined. To accurately investigate motor axon mRNA composition and its modulation in ALS we developed Axon-seq, an application of our spatial single-cell RNA-sequencing technique (Nichterwitz et al., 2016) to microfluidic devices housing mouse embryonic stem cell (mESC)-derived MNs. In contrast to previous methods, Axon-seq does not require RNA isolation and allows high sensitivity and cost-efficient sequencing from a single microfluidic device. Importantly, Axon-seq utilizes a stringent and sensitive bioinformatic quality control (QC) step that identifies samples containing trace levels of mRNA from undesired cell somas, effectively eliminating all cellular cross-contamination.

## Results

### Axon-Seq Is a Refined Method that Enables RNA Sequencing of Axons from a Single Microfluidic Device

**Figure 1:**
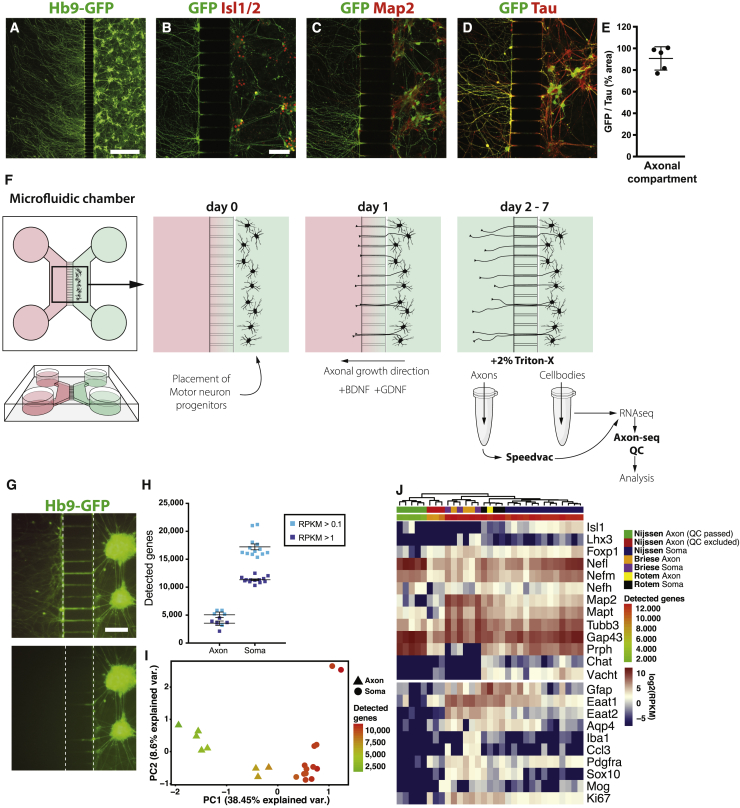
Axon-Seq of Motor Axons in Single Microfluidic Devices (A and B) Anti-GFP immunofluorescence visualizes motor axons crossing the microgrooves and extending into the axonal compartment. MN somas are visualized with (A) Hb9-eGFP and (B) *Isl1/2* staining. (C and D) Presence of Tau (*Mapt*) (D) but very little *Map2* (C) indicates that mainly axons cross over into the other compartment. (E) Quantification of Hb9::eGFP^+^ area over Tau^+^ area reveals that the majority of crossing axons are motor axons (90.75% ± 4.8%). Data are represented as mean ± SEM. (F) Overview of the methodology used. MNs are cultured in one compartment of the microfluidic device and axons are recruited across microgrooves using a gradient of trophic factors. Compartments are separately lysed and cDNA libraries prepared for RNA sequencing. (G) Hb9-eGFP expression reveals that the somatic chamber is not affected by lysis of the axons. (H) The axonal fractions contain around 5,000 unique transcripts, while MN somas contain >15,000 (mean ± SEM). (I) PCA based on all expressed genes showing “contaminated” axon samples with an intermediate number of detected genes that cluster away from the clean axon samples and toward the somatodendritic samples. (J) Expression of selected marker genes in soma and axon samples of this study and data from studies by Rotem et al. (2017) and Briese et al. (2016). Our clean axon samples separate from the rest, while axon and soma samples from previously published studies intermingled, indicating cross-contamination. Scale bars: (A) 500 μm. (B) 100 μm; scale bar in (B) also applies to (C) and (D). (G) 100 μm.

To investigate transcriptional changes in motor axons in health and ALS we used mESC-derived MNs (Wichterle et al., 2002) from a control Hb9-GFP line or cells overexpressing the human mutated SOD1^G93A^ protein. We first confirmed the MN subtype identity of our cultures through bulk RNA sequencing and cross-comparison with single MNs (Nichterwitz et al., 2016). We observed expression of the MN transcription factors (TFs) *Isl1* and *Foxp1* in our cultures (Figure S1A), indicating that MNs of a lateral motor column (LMC) identity were generated (Dasen et al., 2008). mRNAs of the *Hox1–8* families were detected, which define a cervical to thoracic spinal identity as well as brainstem MNs. Expression of *Phox2b* confirmed that a proportion of brain-stem MNs were generated in addition to spinal MNs (Figure S1A). After being plated in microfluidic devices, motor axons were recruited to the vacant chamber by a gradient of glial cell line-derived neurotrophic factor (GDNF) and brain-derived neurotrophic factor (BDNF) (Figures 1A and 1F). An initial concentration of 50 ng/mL of GDNF/BDNF was followed by a lower concentration of 5 ng/mL, once axons had crossed the microchannels, to avoid growth cone collapse (Figure 1A). The HB9-eGFP reporter signal was detectable in motor axons and in somas where it co-localized with Islet1/2 (Figure 1B). We next stained for *Map2* and *Mapt* (*Tau*) (Figures 1C, 1D, and S1B). In mature neurons, *Map2* labels dendrites, while *Tau* labels axons (Garner et al., 1988, Litman et al., 1993). The majority of processes crossing the channels were *Tau* positive, while a minority were *Map2* positive. Thus, axonal processes were enriched compared with dendrites. MN cultures derived from mESCs also contain interneurons. However, since 90.75% ± 4.8% of Tau+ processes were Hb9-GFP+ and only a very low proportion of GFP-negative (and *Tau*-positive) axons appeared in the axonal compartment, we from here on refer to axonal preparations as motor axons.

Adding different volumes to the compartments generates a fluid flow that counteracts diffusion (Mills et al., 2018, Taylor et al., 2005). Using this principle, axonal samples were lysed without affecting the soma compartment (Figure 1G). Each compartment was lysed using 2% Triton X-100 in water, eliminating an RNA purification step. To allow sequencing of individual devices, we performed a vacuum centrifugation concentration step. This allowed using the entire lysate for cDNA library preparation. We then conducted RNA-seq on axonal and soma samples from individual devices using the Smart-seq2 protocol (Picelli et al., 2013) with minor modifications (Nichterwitz et al., 2016).

To be included in bioinformatics analysis, samples required >300,000 mapped reads, >0.4 correlation to at least one other sample, and >2,500 detected genes. We next implemented a crucial bioinformatic quality control (Axon-seq QC), excluding axon samples with trace levels of soma contamination. Here, we performed principal-component analysis (PCA) based on all expressed genes. The PC1 reflected the number of detected genes. Axonal transcriptomes occupied the lower ranges and somatodendritic samples occupied the higher ranges (Figure 1I). Three axonal samples were intermediate, with >7,500 detected genes, and likely contaminated with one or more somas and were discarded. Remaining high-quality axon samples contained ∼3,500 detected genes at >1 reads per kilobase of transcript per million mapped reads (RPKM) and 5,000 detected genes at >0.1 RPKM, while somatodendritic samples contained approximately three times these numbers (Figure 1H). Axonal samples also displayed fewer detected genes compared to single MNs (Nichterwitz et al., 2016) (Figure S1F). Interestingly, despite this difference, the cDNA yields were comparable between axonal samples and single MNs (Figure S1E), suggesting that (1) the cDNA yield from all axons in a microfluidic device approaches that of one or two cells, and (2) low numbers of detected genes in these samples are not a technical artifact, but a trait unique to axons. Given that Axon-seq libraries display lower numbers of detected genes than do single MNs, a single contaminating cell has the potential to drastically alter the readout of an axonal library. We find that consideration of the number of detected genes is perfectly suited to identifying contaminated samples, allowing further downstream analysis on only pure axonal samples.

After QC, we sought to compare our dataset with previously published methods, selecting two RNA-seq studies (Table S1) that used either well insets (Rotem et al., 2017) or single microfluidic devices (Briese et al., 2016) to isolate motor axon transcriptomes. Both these studies display good-quality data and describe enrichment of axonal transcripts. Expression of neuronal markers in all samples was visualized in a heatmap, including, e.g., neurofilaments, peripherin, *Mapt*, *Gap43*, and the glial markers *Gfap*, *Aqp4*, *Eaat1*, *Iba1*, *Ccl3*, *Pdgfra*, *Sox10*, and *Mog* (Figure 1J). This clearly demonstrates that our Axon-seq samples passing QC expressed robust levels of neuronal and axonal markers. It also showed that our samples were devoid of glial markers and the proliferative marker *Ki67*, which were in fact present in all previously published axon sequencing samples (Figure 1J). Furthermore, our axon samples clustered away from somatodendritic samples and our cross-contaminated axon samples, while axon and soma samples from previous studies were intermingled (Figure 1J). Further cross-comparison of our axon samples with data derived from purified neuronal samples (Zhang et al., 2014) confirmed their purity (Figure S1D). Importantly, our Axon-seq samples expectedly displayed a far lower number of detected genes than the previously published axon samples (Figure 1J). Thus, samples displaying even small levels of soma contamination were successfully identified through application of Axon-seq QC.

In summary, our analysis demonstrates the importance of extensive QC of axon transcriptome data with particular consideration for the number of detected genes and presents Axon-seq as a robust and significantly improved method for RNA-seq of axonal fractions from individual microfluidic devices with sensitivity similar to that of single-cell sequencing.

### Axons Have a Unique Transcriptional Profile and Are Particularly Enriched in Transcripts Important for Mitochondrial and Ribosomal Functions

**Figure 2:**
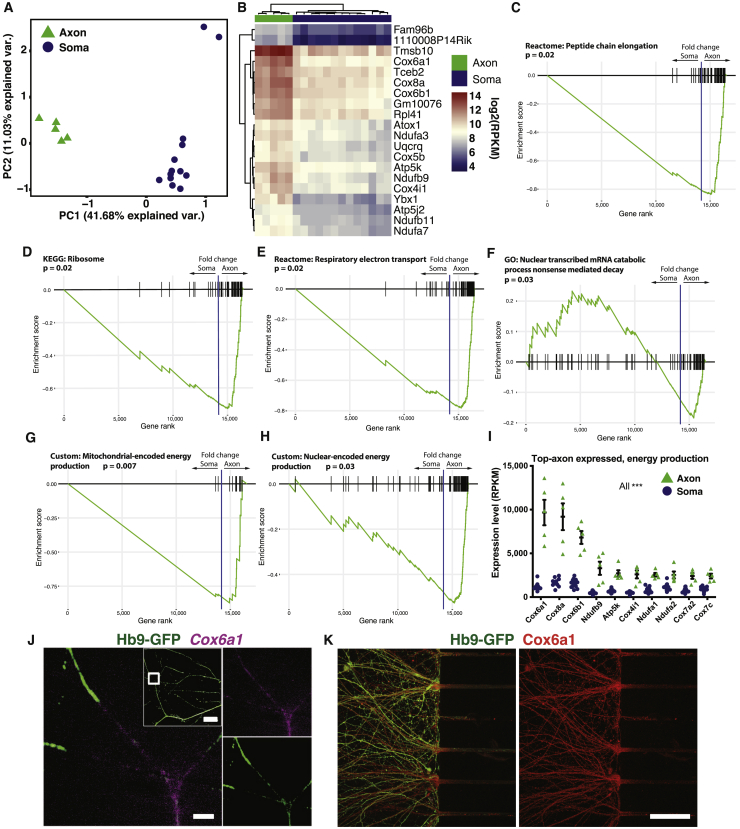
Axons Have a Transcriptome Distinct from that of Somas (A) PCA of all expressed genes reveals a clear separation of axons and somas. (B) Top 20 genes determining PC1-negative loading. All of them are strongly enriched in axons compared with somas. (C–H) Gene set enrichment analysis (GSEA) using fold changes from the DESeq2 differential expression output ranked list between axons and somas, without a p value cutoff. GSEA uncovers pathways related to (C and D) local translation, (E) energy production, and (F) nonsense-mediated decay. (G and H) Subdividing the oxidative energy production class into (G) mitochondrial- and (H) nuclear-encoded transcripts reveals an axonal enrichment for both. Differential expression was performed on the five axon samples that passed QC and 13 soma samples, as visualized in (A) and (B). (I) Expression levels of the highest expressed transcripts in axons related to oxidative energy production. All of the genes plotted are significantly enriched in the axonal compared with the somatodendritic compartment (^∗∗∗^p < 0.001). p values are derived from the DESeq2 differential expression and are adjusted for multiple testing. Data are represented as mean ± SEM. (J) RNAscope and (K) immunocytochemistry of Cox6a1, the most abundant mRNA in axons involved in energy production. Scale bars: (J) 2 μm, lower magnification inset, 20 μm. (K) 50 μm.

We next sought to compare the transcriptome of axonal fractions with that of somatodendritic fractions. When transcripts present in at least 3 of 5 samples were considered, the axonal transcriptome comprised 4,238 genes (Table S2). Between axons and somas, 10,406 genes were differentially expressed. The majority of these (9,762) were soma enriched, while 644 transcripts were axon enriched (Table S2). Moreover, analysis of the top 100 differentially expressed genes in axons and somas clearly demonstrated that axons are not simply diluted somas, but instead have a unique mRNA profile (Figures S1G and S1H). PCA based on all expressed genes clearly distinguished axons from somas, with PC1 explaining 41.7% of the variance (Figure 2A). The top 20 genes causing PC1-negative loading, and thereby axonal identity, included several genes involved in the mitochondrial respiratory chain (*Cox6a1*, *Cox6b1*, *Cox8a*, *Cox4i1*, *Ndufa3*, *Ndufa7*, *Ndufb9*, *Ndufb11*), mitochondrial ATP synthesis (*Atp5k*, *Atp5j2*), ribosome subunits and transcriptional elongation (*Rpl41*, *Tceb2*), cytoskeleton organization (*Tmsb10*), copper delivery (*Atox1*), and mRNA splicing (*Ybx1*) (Figure 2B). PC1-positive loading (Figure 2A) was heavily influenced by transcripts present solely in somas. In total, 2,903 genes expressed at an average RPKM of >1 in somas were undetectable in axons (Figure S1H). Gene set enrichment analysis (GSEA) on axon-enriched genes uncovered pathways related to local translation (Figures 2C and 2D), mitochondrial oxidative energy production (Figure 2E), and nonsense-mediated decay (Figure 2F). Interestingly, both nuclear- and mitochondrial-encoded transcripts were enriched in axons (Figures 2G and 2H), but the 10 most abundant energy production-related transcripts in axons were nuclear-encoded transcripts (Figure 2I).

To confirm axonal localization of particular transcripts we conducted *in situ* hybridization experiments using RNAscope. Negative (*dapB*) and positive (*Ubc*, *Polr2a*, and *Ppib*, poly(A)) control probes demonstrated the specificity of the method (Figures S2A–S2C). We confirmed *Cox6a1* mRNA, which is part of the electron transport chain, to be localized to motor axons (Figure 2J) and somas (Figure S2D). Immunocytochemistry of motor axons demonstrated that also the Cox6a1 protein was readily detected in axons (Figure 2K).

In summary, our data show that axons of growing MNs are enriched for mRNAs related to ribosomes and oxidative phosphorylation, a large majority of which are mitochondrially produced. The distal axon compartment can thereby sustain local translation machinery and high energy metabolism, reflecting the enormous energy demands of growing axons.

### Motor Axons Show a Unique Transcription Factor Profile

**Figure 3:**
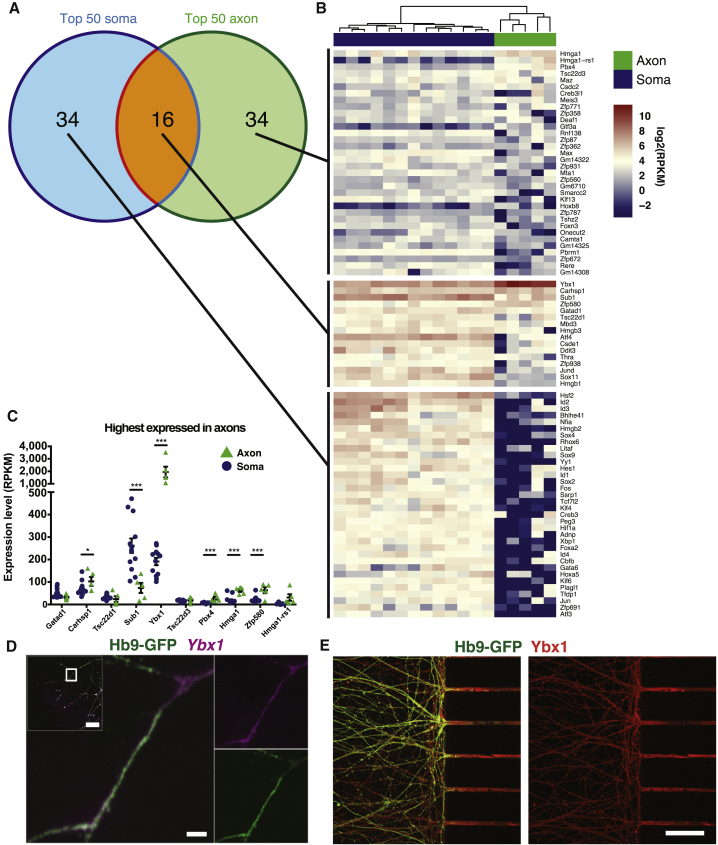
A Unique Transcription Factor Repertoire Localizes to Axons (A) Of the top 50 highest expressed TFs in the soma and axon (at least four axonal samples), 16 overlapped. (B) Heatmap of expression values of TFs from (A). (C) The top 10 TFs with the highest expression in axons, of which five are significantly enriched in axons, while one is enriched in somas (^∗^p < 0.05, ^∗∗∗^p < 0.001). p values are derived from differential expression between the five axon and the 13 soma samples that passed QC and are corrected for multiple testing (mean ± SEM). (D and E) (D) RNAscope and (E) immunocytochemistry for Ybx1, the most abundant TF at the mRNA level in axons. Scale bars: (D) 2 μm, lower magnification inset, 20 μm. (E) 50 μm.

Analysis of the top 50 most prevalent TF mRNAs in each of the two compartments uncovered 16 common TFs, including *Ybx1*, *Carhsp1*, and *Sub1* (Figures 3A and 3B). The majority of soma-enriched TF mRNAs were more abundant in somas than the most axon-enriched TFs were in axons (Figure 3B). However, 5 of the 10 most abundant axonal TF mRNAs were significantly enriched in this compartment compared to somas, including *Ybx1*, *Carhsp1*, *Pbx4*, *Hmga1*, and *Zfp580* (Figure 3C). The enrichment of *Ybx1* (Y-box binding protein 1, p < 0.001) (Figure 3C) was particularly compelling since Ybx1 partakes in, e.g., pre-mRNA transcription and splicing, mRNA packaging, and regulation of mRNA stability and translation (Lyabin et al., 2014a, Lyabin et al., 2014b). RNAscope confirmed the presence of *Ybx1* mRNA in Hb9-GFP+ motor axons (Figure 3D). Immunocytochemistry demonstrated that also the Ybx1 protein was localized to motor axons (Figure 3E), strengthening a possible role for Ybx1 in supporting local regulation of distal axonal processes.

In summary, axons show a unique TF profile, reflecting specific functions in this compartment, that could potentially function as communication signals between somas and axons.

### Cross-Comparison of Axonal Datasets across Neuronal Subtypes Reveals Both Motor Neuron-Enriched and Pan-Neuronal Axon mRNA Repertoires

To understand which mRNAs are specific to motor axons versus transcripts defining growing axons in general, we compared our Axon-seq motor axon data with a published axon dataset derived from primary embryonic mouse MNs (Briese et al., 2016), and with primary embryonic mouse dorsal root ganglia (DRG) (Minis et al., 2014). In both comparisons, Gene Ontology (GO) term analysis on the overlapping genes revealed oxidative energy production, translation, and localization of proteins to mitochondria and ribosomes (Figures S3A and S3B, Table S3). Both primary motor axon and primary DRG axon datasets contained a large number of proliferative and glial marker sets that were absent from our Axon-seq data (Figures 1J and S3C). This was further confirmed by GO term analysis of the transcriptomes unique to Briese et al. and Minis et al., which in both cases identified biological processes involved in cell division (Figures S3A and S3B, Table S3).

Notably, *Cox6a1* and *Ybx1*, abundant mRNAs in motor axons, were present across neuron types. Furthermore, a low-expressed, but important transcript, *Nrp1*, was also pan-neuronal (Figures S3A and S3B). We could confirm the presence of this transcript in our motor axons using RNAscope (Figure S3D).

In summary, our analyses have identified a general axonal transcriptional signature and defined a unique motor axon code, which gives clues to function as well as susceptibility to disease.

### Axon-Seq of Human Motor Axons Identifies a Common Transcriptome with Mouse Motor Axons

**Figure 4:**
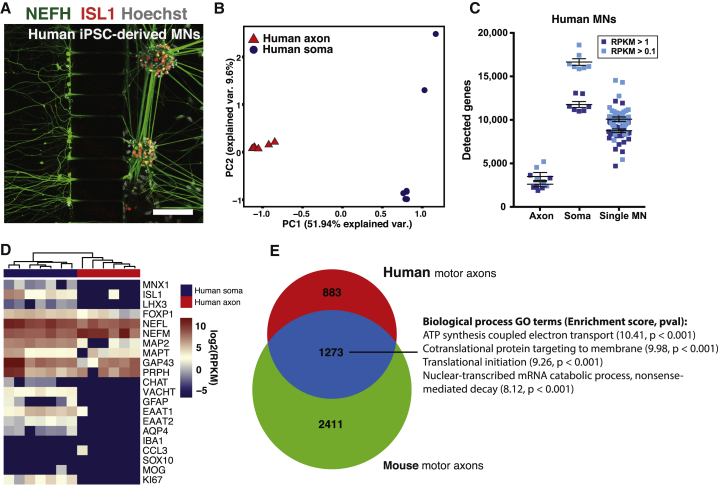
Human Motor Axons Have a Distinct Transcriptome that Overlaps with Mouse Motor Axons (A) Immunocytochemistry of human control iPSC-derived MNs growing in a microfluidic device. (B) PCA based on all expressed genes shows clear separation of human axonal and somatodendritic fractions along PC1. (C) Numbers of detected genes in human bulk somas, axons, and single human Hb9-GFP+ MNs. Human axons contain >3,400 transcripts, while somas contain >15,000 transcripts at RPKM >0.1. Single cells are intermediate with approximately 10,000 transcripts detected. Data are represented as means ± SEM. (D) Expression of selected marker genes in human soma and axon samples reveals strong axonal presence of mRNAs for cytoskeletal elements and the TF *FOXP1*. Glial markers are absent in axons. (E) Comparison of the human motor axon transcriptome with the common mouse motor axon transcriptome (Figure S3A) reveals an overlap of 1,273 genes (after exclusion of non-orthologous genes). These genes enrich in GO terms for energy production, translation, and nonsense-mediated decay. Scale bar: (A) 100 μm.

We further applied Axon-seq to human MNs derived from two control induced pluripotent stem cell (iPSC) lines. Human motor axons could be readily recruited across microgrooves using a BDNF/GDNF gradient (Figure 4A). In a PCA plot, axonal samples clearly separated from human somatodendritic fractions (Figure 4B). Considering transcripts present in at least three axonal samples, the human motor axonal transcriptome comprised 2,793 genes (Table S4). The number of detected genes was on average 2,611 ± 286 at RPKM >1, and 3,495 ± 460 at RPKM >0.1 (Figure 4C). Human motor axons were strongly enriched for cytoskeletal transcripts such as *NEFL*, *NEFM*, and *GAP43* and showed negligible glial contamination (Figure 4D).

Analysis of the overlap between the human motor axon transcriptome and the common mouse motor axon transcriptome (Figure S3A) identified 1,273 common genes. GO term analysis of these genes revealed energy production, translation, and nonsense-mediated decay as enriched processes (Figure 4E, Table S4).

### The ALS-Causative Mutation SOD1^G93A^ Modulates the Axonal Transcriptome

**Figure 5:**
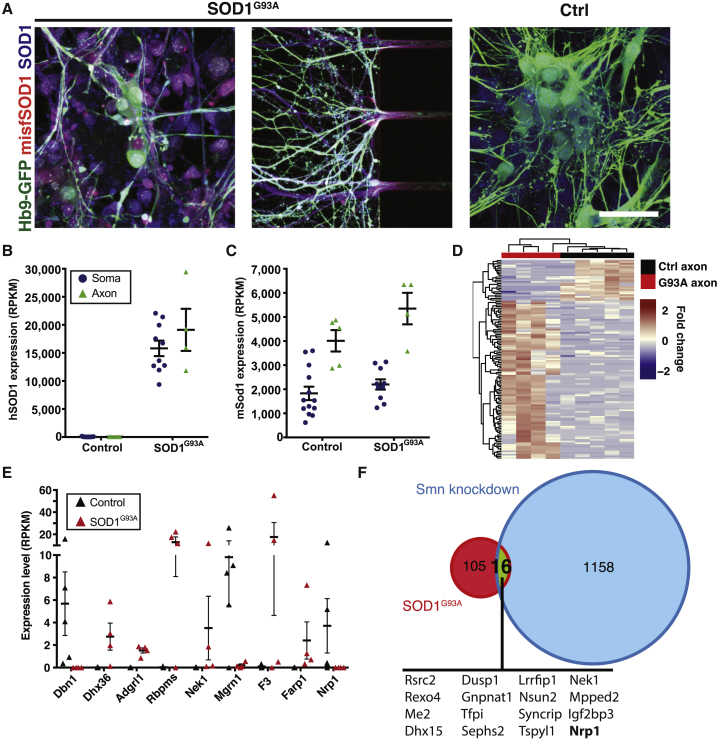
Overexpression of SOD1^G93A^ Induces Changes in the Axonal Transcriptome (A) Staining for SOD1 and misfolded SOD1 in MN cultures. In SOD1^G93A^-expressing cells high levels of both SOD1 and misfSOD1 are detected, whereas no misfSOD1 is detected in control cells. (B and C) (C) Mouse *Sod1* is enriched in axons, while (B) transgenic human *SOD1* is equally divided over both compartments. Levels of *hSOD1* are approximately 10-fold higher in somas than those of mouse *Sod1*. Data are represented as means ± SEM. (D) Heatmap of differentially expressed genes between five control axon samples and four axon samples from SOD1^G93A^-overexpressing MNs. A total of 121 genes are differentially expressed, of which 25 are enriched in control axons and 96 in SOD1^G93A^ axons. (E) Selected differentially expressed genes from the analysis visualized in (D) with relevance to ALS and general neuronal functioning (all p < 0.05). All p values are derived from the DESeq2 output and are corrected for multiple testing (mean ± SEM). (F) Overlap between dysregulated genes upon SOD1^G93A^ expression in our dataset and Smn knockdown in data from Saal et al. (2014). Sixteen genes are commonly dysregulated. One gene, Nrp1, is commonly downregulated. Scale bar: (A) 50 μm.

Motor axons are early pathological targets in the lethal MN disease ALS, where die-back pathology begins in the distal axon. We therefore investigated if overexpression of an ALS-causative mutation (*SOD1*^*G93A*^) would induce changes in the axonal transcriptome. MNs derived from mESCs overexpressing human SOD1^G93A^ have high levels of SOD1 protein in somas and axons (Figure 5A). Axon-seq of SOD1^G93A^ axons identified 4,479.5 ± 1,327.7 genes at RPKM >1 and 6,568.8 ± 2,293.1 at RPKM >0.1 (Table S5). Human mutant *SOD1* was highly expressed in somas and axons (Figure 5B), 10-fold higher than mouse *Sod1* (Figure 5C). Mutant *SOD1* overexpression resulted in differential expression of 121 genes in axons compared to the control line, of which 96 were upregulated in SOD1^G93A^ axons, while 25 were reduced (Figure 5D, Table S5). Strikingly, only two of these genes (*Zfand1* and *Zfp688*) were also dysregulated in the SOD1^G93A^ soma compartment (Table S5). Many of the dysregulated genes were previously implicated in neuronal function and in ALS pathology (Figure 5E). We found that several transcripts paramount for proper axon function were downregulated or completely absent in SOD1^G93A^ axons, including *Dbn1* (McIntyre et al., 2010, Sharma et al., 2012), *Nrp1* (Telley et al., 2016), and *Mgrn1* (Upadhyay et al., 2016). Some transcripts that were upregulated in SOD1^G93A^ axons appear detrimental, including *Adgr1* (Zuko et al., 2016) (Figure 5E). In addition, upregulation of some transcripts in ALS could reflect compensatory mechanisms preventing axon damage or dysfunction in SOD1^G93A^ axons, including *Dhx36* (Bicker et al., 2013), the ALS-causative gene *Nek1* (Kenna et al., 2016), *F3* (Bizzoca et al., 2009), *Rbpms* (Hornberg et al., 2013), and *Farp1* (Zhuang et al., 2009) (Figure 5E).

Finally, we wanted to elucidate if any of the transcripts that were dysregulated by the SOD1^G93A^ mutation were affected across MN diseases. We therefore compared our RNA sequencing data with a microarray dataset on motor axons in which the *Smn* gene was knocked down to model the disease spinal muscular atrophy (SMA) (Saal et al., 2014). Sixteen transcripts were regulated across the two disease models. However, 15 genes were regulated in the opposite direction in the ALS and SMA disease models, while one transcript, the axon guidance receptor Neuropilin 1 (*Nrp1*), was downregulated in both MN disease models (Figure 5F).

In summary, application of Axon-seq methodology to a mutant *SOD1* overexpression model of ALS identified axonal dysregulation of 121 transcripts, some of which appear detrimental, while others may indicate compensatory mechanisms to counteract any induced dysfunction. *Nrp1* was downregulated in motor axons across ALS and SMA disease models and might have implications for pathology.

## Discussion

Axonal RNA biology is increasingly implicated in ALS. We developed Axon-seq, an improved method for studying the transcriptome of axons. With careful bioinformatic QC, this cost-effective and sensitive method can reliably yield high-quality and pure axonal transcriptomes. Axon-seq was successfully applied to mouse and human MNs from multiple pluripotent sources, cultured in microfluidic devices. We detected 3,500–5,000 mRNA transcripts in axons, which were enriched for mitochondrial energy production and ribosomes, reflecting a high energy dependence of growing axons and the importance of local protein translation. Indeed, protein synthesis in chick sympathetic axons accounts for 5% of total cellular protein synthesis (Lee and Hollenbeck, 2003). Transcripts for all essential subunits of the proteasome system (*Psma1–7* and *Psmb1–7*) were present in motor axons (Table S2). Many regulatory subunits and chaperones were also detected, implying extensive regulation of protein degradation in axons. Given that small amounts of RNA can be translated into multiple protein copies, maintaining an mRNA pool poised for translation in neuronal processes saves energy and increases capacity for a rapid response.

The two largest mRNA groups identified in axons through GO term analysis and GSEA were mitochondrially encoded mRNAs involved in energy production and mitoribosomes. As mitochondria are abundant in axons, and assuming no bias in soma to axon transport of mitochondrially encoded RNAs, we speculate that these mRNAs are locally transcribed. This is consistent with our finding that nuclear-encoded mRNAs for energy production (in particular nuclear-encoded ribosomal RNAs) were less abundant in axons compared with mitochondrially encoded genes and in agreement with previous reports describing a predominance of mitochondrially encoded genes in DRG axons (Minis et al., 2014). Mitochondria can replicate by fission in axons (Amiri and Hollenbeck, 2008), which would be facilitated by local translation of mitochondrially encoded proteins. In fact, our data suggest that the complete mRNA library required for mitochondrial replicative fission and function is present in axons.

TFs define neuronal identity in early development and are later often needed for survival (Kadkhodaei et al., 2013, Sgado et al., 2006). Some TFs were recently shown to function outside the soma and to be important in neuronal circuitry plasticity, axon pathfinding, and neuroprotection (Sugiyama et al., 2008, Torero Ibad et al., 2011, Wizenmann et al., 2009). Multiple studies identified axonal mRNAs for TFs, in addition to nuclear transport machinery and the nuclear envelope, whose local translation and subsequent signaling to the soma appear vital to axon maintenance and survival in injury paradigms (Ben-Yaakov et al., 2012, Cox et al., 2008, Hanz et al., 2003, Ji and Jaffrey, 2012, Yoon et al., 2012). We detected a specific set of TF mRNAs enriched in distal motor axons compared with somas, including the RNA-binding factor *Ybx1*. YBX1 has roles in binding and stabilizing cytoplasmic mRNAs. It also regulates translation by affecting the interaction between eukaryotic initiation factors and mRNAs (Lyabin et al., 2014b). YBX1 can also mediate anterograde axonal transport of *Cox4* mRNA, encoding a mitochondrial protein (Kar et al., 2017). In this function YBX1 interacts with FUS, an RNA binding protein and known ALS-causing gene (Groen et al., 2013). We expect that YBX1 and other axonally located TFs will emerge as important mediators of communication between somas and axons.

Dying-back pathology in vulnerable MNs implies that the distal axonal compartment undergoes early pathological changes in ALS. Axon-seq on motor axons harboring the ALS-causative mutation SOD1^G93A^ uncovered differential expression of 121 mRNAs, several of which are crucial for neuronal function, axon maintenance, and growth. For example, a number of the transcripts that were upregulated in SOD1^G93A^ axons appear detrimental, including *Adgr1*, which can induce apoptosis and cause reduction in neurite outgrowth (Zuko et al., 2016). Multiple transcripts that are important for axon function were downregulated or absent in SOD1^G93A^ axons, including *Dbn1*, which is important for axon initiation, growth, and guidance (McIntyre et al., 2010, Sharma et al., 2012); *Nrp1*, a semaphorin receptor involved in both axon guidance and subcellular target recognition (Telley et al., 2016); and *Mgrn1*, a ubiquitin ligase, which appears important for mitochondrial function and neuronal survival (Upadhyay et al., 2016). *Mgrn1* is downregulated in SOD1 mice and is recruited to SOD1-positive inclusions (Chhangani et al., 2016). *Nrp1* acts as an axonal attractant during development (Chauvet et al., 2007) and is important for limb innervation (Huettl et al., 2011). The downregulation of *Nrp1* in our study appears to align with a loss of NMJ function. Nonetheless, it was recently shown that intraperitoneal delivery of an anti-Nrp1 antibody to SOD1^G93A^ mice could lengthen their lifespan and reduce NMJ denervation (Venkova et al., 2014). Although it is not evident if downregulation of Nrp1 within MNs induced the rescue in the Venkova et al. study, this could mean that the downregulation we see may be a beneficial compensatory event, but this remains to be further investigated.

*Nrp1* was downregulated in motor axons across SMA and ALS disease models. While SMA is generally an early-onset MN disease and ALS is a disease correlated with aging, both are characterized by early motor axon pathology (Cifuentes-Diaz et al., 2002, Comley et al., 2016, Fischer et al., 2004). Our data combined with published studies indicate that NRP1 might be an early target in ALS pathology and that it potentially could be modulated in both diseases with beneficial results.

Interestingly, the SOD1 mutation caused an upregulation of transcripts that could be beneficial to axons, possibly through compensatory mechanisms aimed at preventing axon damage or dysfunction. These transcripts with potential beneficial properties included *Dhx36*, an ATP-dependent helicase, which mediates dendritic localization of miR-134, affecting synaptic protein synthesis and plasticity (Bicker et al., 2013); *F3*, a cell adhesion molecule involved in neurite outgrowth (Bizzoca et al., 2009); *Rbpms*, an RNA-binding protein with importance for RNA-granule localization and dendritic tree complexity (Hornberg et al., 2013); *Farp1*, a marker for LMC MNs that positively regulates dendrite growth (Zhuang et al., 2009); and *Nek1*, which has a putative role in microtubule stability, neuronal morphology, and axon polarity. Interestingly, loss-of-function variants of *NEK1* confer susceptibility to ALS in humans (Kenna et al., 2016).

In summary, we have developed Axon-seq, a refined method with high sensitivity for RNA sequencing of axons, which contains crucial bioinformatic QC steps to identify high-quality axonal transcriptomes. We demonstrate that mouse and human motor axons contain a smaller and distinct transcriptome compared to somas, with high enrichment for transcripts required for local energy production and protein translation. We also show that the majority of motor axonal genes are shared with axons of other neuronal types, emphasizing their importance in general axonal biology. Finally, motor axons expressing ALS-causative SOD1^G93A^ displayed both down- and upregulation of key transcripts involved in axonal growth and guidance, as well as neuronal survival.

Due to its robustness, sensitivity, and cost effectiveness, Axon-seq is a method of broad utility for the study of RNA dynamics in any polarized cell. Here we successfully applied Axon-seq to human motor axons, demonstrating its usefulness for the study of human neuronal processes in stem cell-derived *in vitro* systems. Thus far, axons of human stem cell-derived glutamatergic neurons have been isolated in microfluidic devices and screened using microarrays (Bigler et al., 2017), but not using more advanced RNA-seq methods. Microfluidic devices have also been used to investigate axonal trafficking deficits and axonal degeneration in ALS patient stem cell-derived MNs (Naumann et al., 2018). Here, Axon-seq can provide an additional layer of transcriptome data, facilitating identification of networks underlying pathological changes in human motor axons. In our system, we studied growing axons prior to formation of NMJs. Stage-specific changes in the axonal mRNA repertoire were recently identified in growing, pruning, and mature axons in mouse retinal ganglion cells (Shigeoka et al., 2016). It is expected that also the motor axon transcriptome is influenced by the formation and maturity of the NMJ. Axon-seq could be applied to a microfluidic system containing iPSC-derived MNs and muscle to investigate such processes in the context of the human NMJ and its instability in ALS.

## Experimental Procedures

### Culturing of Mouse Motor Neurons in Microfluidic Devices

Microfluidic devices (SND150, Xona Microfluidics) were attached to sterilized cover glasses (28 mm diameter, Menzel Gläser) by drying them extensively and applying gentle force to seal them onto the glass coverslips. Both compartments were coated overnight with 15 μg/mL poly-L-ornithine (Sigma-Aldrich). The secondary coating consisted of a mix of 2 μg/mL fibronectin (Sigma-Aldrich) and 10 μg/mL laminin (Sigma-Aldrich).

MNs were resuspended at 100,000 cells/μL and loaded into the microchannel in a 5 μL droplet. At this point, neural differentiation medium was supplemented with 10 ng/mL GDNF, BDNF, ciliary neurotrophic factor, and neurotrophin-3; 200 μM ascorbic acid (Sigma-Aldrich); 100 nM retinoic acid (RA); and 2 μM 5-fluoro-2′-deoxyuridine (Sigma-Aldrich). Cells were allowed at least 30 min to attach, after which medium was added into the adjacent wells. The axonal compartment was filled with similar medium but containing 50 ng/mL GDNF and BDNF to facilitate axonal recruitment. Volumes in the wells were adapted to ensure flow across chambers to gradually provide medium and to establish a flow and thus trophic factor gradient from the axonal to the somatic compartment. Media in the devices was changed daily.

Axonal recruitment was halted after 2 days. The concentrations of GDNF and BDNF in the axonal compartment were reduced to 5 ng/mL. In the soma compartment, trophic factor concentrations were doubled from this point onward, to 20 ng/mL. Additionally, RA was no longer included in the media.

### Culturing of Human Motor Neurons in Microfluidic Devices

The use of human stem cell lines was approved by the regional ethical review board in Stockholm, Sweden (Regionala Etikprövningsnämnden (EPN), Stockholm). For human MNs, devices with 450 μm grooves were used (SND450, Xona Microfluidics). The procedures were largely similar to those for culturing mouse MNs in microfluidic devices, with the following exceptions. Human MNs were plated at a lower density of 30,000 cells/μL (150,000 cells/device). After dissociation, cells were plated in devices in B27 medium supplemented with 5 μM Rock inhibitor, 200 μM ascorbic acid, and 10 μM DAPT. BDNF and GDNF (10 ng/mL) were added to the somatodendritic compartment, while 50 ng/mL BDNF and GDNF were added to the axonal compartment for recruitment of motor axons. After 3 days in the devices, DAPT was removed from the medium. BDNF and GDNF levels were reduced to 10 ng/mL in the axonal compartment.

### Harvesting, Library Preparation, and Sequencing

Cultures were harvested after 1 week (mouse) or 2 weeks (human) in the microfluidic devices using 2% Triton X-100 (in water) and 1.5 U of RNase inhibitor (TaKaRa). Lysis solution (75 μL) was flushed through the compartments, collected, and snap-frozen on dry ice. Prior to library preparation, the volume of axonal samples was reduced from 75 to 15 μL using a concentrator (Eppendorf), and 10 μL of the lysate was used for reverse transcription. Five microliters of the lysates from somatodendritic samples was directly used for the reverse transcription reaction, carried out in a final volume of 10 μL. Further steps were carried out as described (Nichterwitz et al., 2016) and samples sequenced on Illumina HiSeq2000 and 2500 platforms.

### RNA-Seq Data Processing and Analysis

Sequencing reads were mapped to the mm10 (mouse) and the hg38 (human) reference genome with HISAT version 2.0.3 (Kim et al., 2015). Aligned reads were extracted and assigned using the GenomicAlignments package (version 1.8.4, Lawrence et al., 2013) in R, with the function summarizeOverlaps, mode set to “union.” QC exclusion criteria were <300,000 (axons) or <500,000 (somas) uniquely mapped reads, Spearman correlation to other samples <0.4 or <2,500 genes expressed at RPKM >0.1. To ensure that high-quality axonal samples were not biologically cross-contaminated with material from cell somas, during the cell seeding process or the lysis, we conducted Spearman correlation, unsupervised hierarchical clustering, and PCA of all samples that passed the initial QC. Axonal samples that clustered with soma samples in these analyses were removed. For the control Hb9-GFP mESC line, MN soma samples (n = 13) and axon samples (n = 8) were derived from a total of six independent experiments. For the SOD1^G93A^-overexpressing mESC line, MN soma samples (n = 10) and axon samples (n = 4) were derived from a total of six independent experiments. Human MN soma samples (n = 7) and axon samples (n = 6) were derived from two iPSC lines. The human single MN analysis was based on n = 39.

## Author Contributions

E.H. conceived the project. J.N., J.A.B., and E.H. designed the experiments. J.N., J.A.B., and R.H. acquired data. J.N., J.A.B., R.H., N.K., and E.H. analyzed data. E.H. supervised the project. J.N. and E.H. wrote the manuscript with the help of J.A.B. and N.K. All authors edited and gave critical input on the manuscript.

## Accession Numbers

The original RNA sequencing data from this study is available at the NCBI Gene Expression Omnibus under accession number GEO: GSE121069.

## Supplemental Information

Document S1. Supplemental Experimental Procedures and Figures S1–S3

Table S1. Details the Antibodies Used in the Study, as well as the RNA-Seq Studies Used for Cross-Comparison in the Manuscript and Accompanies Data Presented in Figures 1, 2, 3, 4, and 5

Table S2. Mouse Axonal Transcriptome contains the mouse motor axonal transcriptome, as defined by genes detected in three of five axonal samples, as well as the differentially expressed genes between mouse control axons and somas and accompanies Figures 1, 2, and 3.

Table S3. Mouse Axonal RNA-Seq Datasets Cross-Comparison contains the gene lists corresponding to the Venn diagrams in Figures S3A and S3B, showing comparisons of our mESC-derived motor axon transcriptome with primary motor axons (Briese et al., 2016) and primary DRG axons (Minis et al., 2014), as well as the associated full GO term analysis sheets.

Table S4. Comparison of Mouse and Human Motor Axonal Transcriptome contains the human motor axonal transcriptome, as defined by genes detected in three of six axonal samples, which accompanies Figure 4, as well as the full GO term analysis sheet of the overlapping genes between the mouse and the human motor axon transcriptome, accompanying the Venn diagram in Figure 4E.

Table S5. Dysregulation of the Mouse Motor Axonal Transcriptome in ALS contains the list of genes that are differentially expressed between mouse SOD1^G93A^ somas and axons, with the associated fold change, p value, and RPKM levels in all samples. It also contains the differentially expressed genes between control and SOD1^G93A^ axons, as well as the RPKM values of these 121 genes in the soma compartments of both lines.

Document S2. Article plus Supplemental Information
