# Supplementary material for: Axon-Seq Decodes the Motor Axon Transcriptome and Its Modulation in Response to ALS
Source: Stem Cell Reports. 2018 Dec 11;11(6):1565–78. doi: 10.1016/j.stemcr.2018.11.005 (PMC6294264; doi:10.1016/j.stemcr.2018.11.005)
Supplement: Document S1. Supplemental Experimental Procedures and Figures S1–S3 [file mmc1.pdf]

**Stem Cell Reports, Volume *11***

## Supplemental Information

# Axon-Seq Decodes the Motor Axon Transcriptome and Its Modulation in Response to ALS

**Jik Nijssen, Julio Aguila, Rein Hoogstraaten, Nigel Kee, and Eva Hedlund**

**SUPPLEMENTAL INFORMATION**

**Axon-seq decodes the motor axon transcriptome and its modulation in response to ALS**

*Jik Nijssen[1,3], Julio Aguila[1,3], Rein Hoogstraaten[1,2], Nigel Kee[1] and Eva Hedlund[1]**

*[1] Department of Neuroscience, Karolinska Institutet, Stockholm, 171 77, Sweden*

*[2] Department of Translational Neuroscience, Brain Center Rudolf Magnus, UMC Utrecht, 3984 CG Utrecht, Netherlands, [3] Co-first authors, * Corresponding author*

**Supplemental Figure S1. Characterization of motor neuron cultures and quality control of axonal samples. (A)** Expression of Hox transcription factors, as well as *Isl1*, *Foxp1* and *Phox2b* in the somatodendritic bulk samples and in single selected Hb9::eGFP+ motor neurons. **(B)** Red-channel images from immunostainings from Fig. 1C and D show clear enrichment for Tau+ neurites, but not Map2+ neurites in the axonal compartment, implying an enrichment for axons. **(C)** Hoechst-negative axonal swellings occur at the groove exits in the axonal compartment. **(D)** Expression of selected marker genes in soma and axon samples, compared with single selected Hb9::eGFP motor neurons and sequencing data of purified glial cell types derived from *Zhang et al.* 2014. **(E)** cDNA yield of axonal samples compared with somatodendritic bulk samples and single cell samples revealed that axonal samples contained a similar amount of cDNA after library preparation as single cell sequencing libraries. Data is represented as mean ± SEM. **(F)** Number of detected genes in axon and soma samples, as well as single isolated motor neurons. Even though axons have similar levels of cDNA yield after library preparation, they have a lower number of detected genes compared to single motor neurons. Data is represented as mean ± SEM. **(G)** Heatmap of 200 differentially expressed genes, the top 100 enriched in soma and the top 100 enriched in axon by p-value. **(H)** Heatmap of all transcripts that were not detected in axons, but present at an average of > 1 RPKMs in somas (n = 2,903 genes). Scale bar: **B:** 50 µm.

**Supplemental Figure S2. Validation of controls for RNAscope and somatic RNAscope for Ybx1 and Cox6a1. (A)** The negative control RNAscope probe against a bacterial gene showed no signal. **(B)** The positive control probes for three housekeeping genes showed positive signals. **(C)** A poly-A probe revealed all poly-adenylated RNAs. RNAscope of **(D)** *Ybx1* and **(E)** *Cox6a1* demonstrated their presence in somas and axons of Hb9::GFP motor neurons. Scale bars: **A:** 20 µm. **B:** 20 µm. **C:** 10 µm, higher magnification inset: 15 µm. **D:** 20 µm, higher magnification inset: 5 µm (*Ybx1*), 2 µm (*Cox6a1*).

**Supplemental Figure S3. Cross comparison with primary mouse motor axon and DRG axon datasets. (A)** Cross comparison of the Axon-seq data with the primary motor axon dataset from *Briese et al*. (2016) **(B)** and the primary DRG axon dataset from *Minis et al*. (2014) revealed that a majority of the transcripts identified with Axon-seq overlapped with the other data sets. **(A,B)** GO terms enriched across data set included oxidative energy production and local translation. Both primary datasets contained non-overlapping genes that enriched for GO-terms related to cell division, implying possible contamination with cell somas of dividing cells. **(C)** Expression of selected marker genes in the dataset from Minis et al. (2014) revealed enrichment for neurofilaments, but also the presence of glial markers

in both soma and axonal compartments. **(D)** Lowly expressed, but biologically important, transcripts in axons e.g. *Nrp 1*, can be detected using RNAscope. Scale bars: **D:** 10 µm, higher magnification inset: 15 µm.

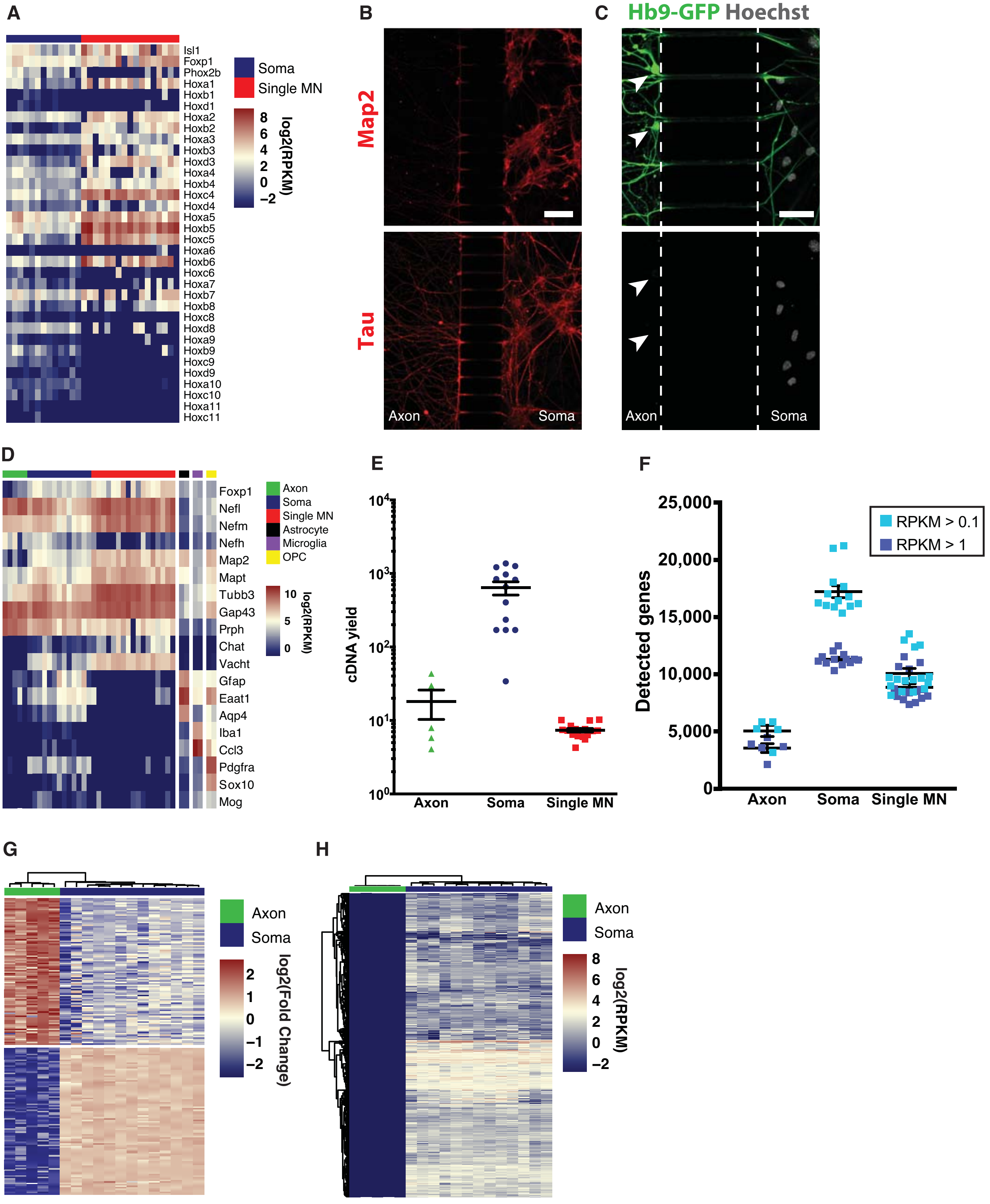


Supplemental Figure S1

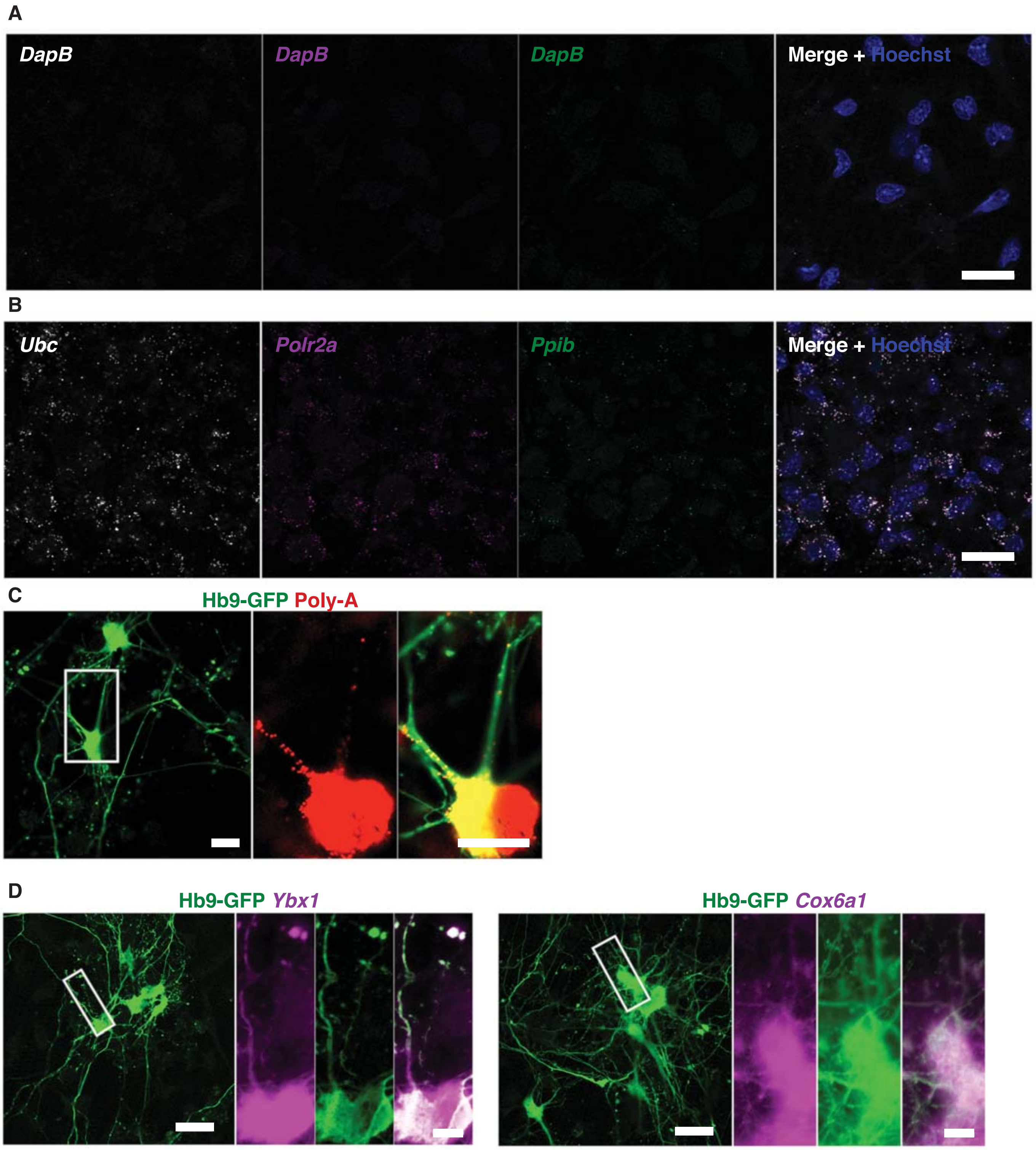


**Supplemental Figure S2**

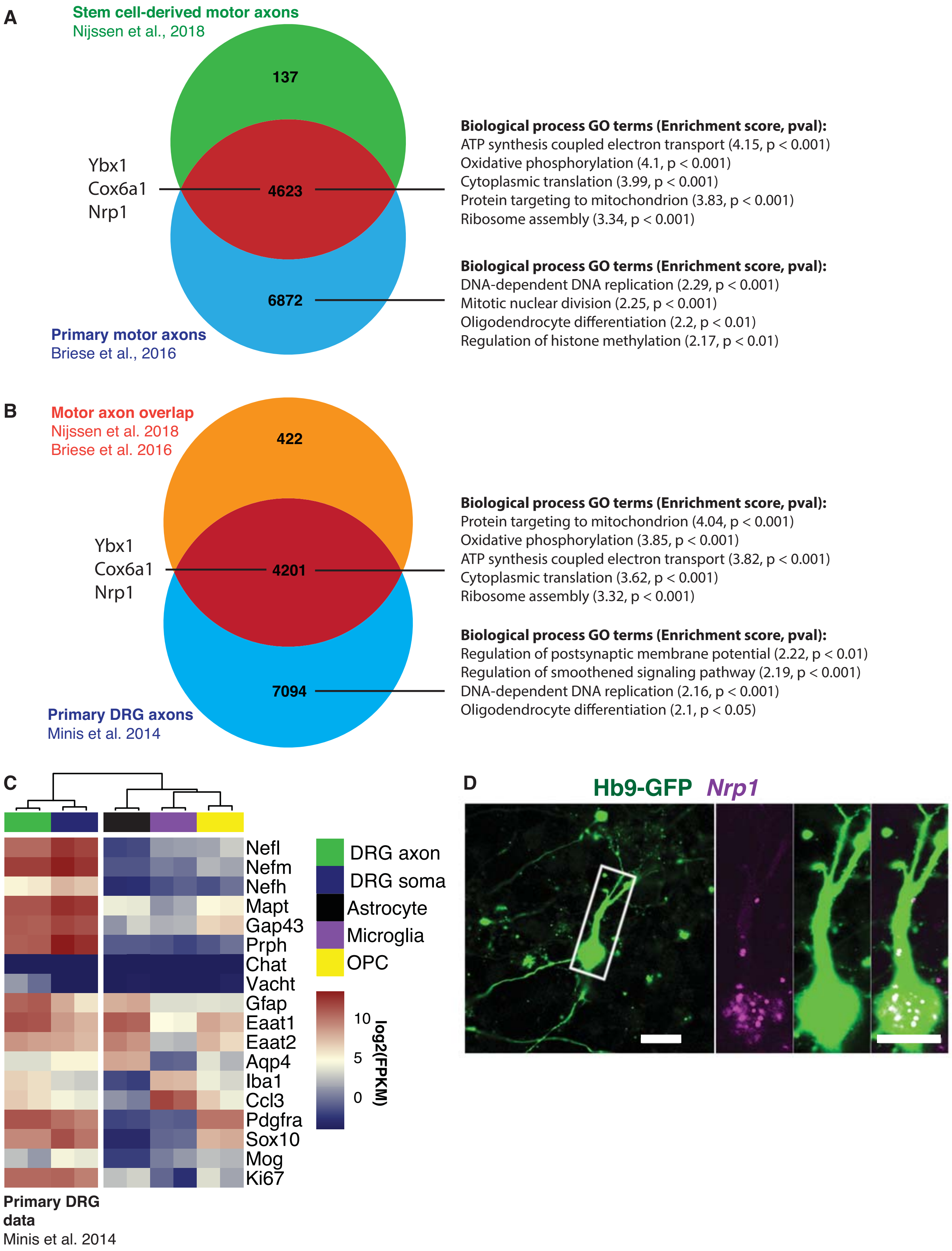


**Supplemental Figure S3**

## Supplemental Experimental Methods

### Mouse embryonic stem cell lines

We used two mouse embryonic stem cell (mESC) lines that express GFP under the Hb9 promoter. One of the lines overexpressed human *SOD1*$^{G93A}$ (passage 12), while the other line expressed only the Hb9::GFP reporter (passage 14). Both lines were derived from Hb9::GFP/SOD1$^{G93A}$ mice and generously provided by Dr. Sebastian Thams (Thams et al., 2018).

### Differentiation of mouse embryonic stem cells into motor neurons

mESCs were expanded on tissue culture dishes coated with 0.1% gelatin (Sigma-Aldrich) in ES media for two days: Knock-Out DMEM with 15% Knock-Out serum, 2mM L-glutamine, 100nM non-essential amino acids, 100 U/ml each of penicillin and streptomycin, 80nM β-mercaptoethanol (all Thermo Fisher) and $10^6$ U/ml leukaemia inhibitory factor (LIF, Merck-Millipore). Then, mESCs were dissociated with TrypLE Express (Thermo Fisher) and purified for 30 minutes on gelatin-coated plates. Embryoid body formation was conducted for 2 days by culturing mESCs in suspension ($4x10^5$ cells/ml media) in neural differentiation media: 1:1 mix of Neurobasal and DMEM/F12 (Thermo Fisher) supplemented with 1x B27 (Thermo Fisher) 2mM L-glutamine, 100nM β-mercaptoethanol and 100 U/mL each of penicillin and streptomycin. During the initial day of embryoid body formation the media was kept in constant swirling motion at 30 rpm to ensure that uniformly sized spheres were formed. Embryoid bodies were subsequently patterned by the addition of 500nM smoothened agonist (SAG, R&D) and 100nM retinoic acid (RA, Sigma-Aldrich) to the neural differentiation media for four days (Nichterwitz et al., 2016). Embryoid bodies containing motor neurons were dissociated for 20 minutes using TrypLE Express (Thermo Fisher), filtered through a 40 μm cell strainer (Fisher Scientific) and spun down for 5 minutes at 1,200 rpm for cell plating into the microfluidic devices.

### Human induced pluripotent stem cell lines

Two control human iPSC lines were used for motor neuron differentiations in microfluidic devices. One iPSC line was purchased from WiCell (DF6-9-9T.B, passage 17). The second iPSC line (39b-corrected, passage 25) was generously provided by Prof. Kevin Eggan (Kiskinis et al., 2014). A third human embryonic stem cell line expressing GFP under the murine Hb9 promoter was used for isolating single GFP+ motor neurons. This line was generously provided by Prof. Kevin Eggan (Di Giorgio et al., 2008).

**Differentiation of human pluripotent stem cells into motor neurons**

Human embryonic stem cells and induced pluripotent stem cells were maintained on Matrigel-coated culture dishes (Corning) and passaged with 5 µM Rock-inhibitor (Y-27632; Tocris). For differentiation into motor neurons, the protocol reported in (Guo et al., 2017) was used with minor adaptations. Briefly, cells were dissociated with TrypLE Express and resuspended as single cells in N2/B27 media to form embryoid bodies (DMEM-F12 supplemented with N2 in a 1:1 ratio with Neurobasal media supplemented with B27, all Thermo Fisher). For the first two days, this media was further supplemented with 5 µM Rock-inhibitor, 40 µM SB-431542, 200 nM LDN-193189 and 3 µM CHIR99021 (all Tocris) and 200 µM ascorbic acid (Sigma-Aldrich). As of the third day, the media was supplemented with 100 nM RA, 500 nM SAG and 200 µM ascorbic acid. On day ten, the EBs were dissociated for plating into microfluidic devices.

**Immunocytochemistry**

For immunocytochemistry cells were fixed in the devices with 4% PFA for 20 min. All subsequent immunocytochemistry procedures and imaging were performed on intact devices.

After fixation, cells were permeabilized with 0.1% triton X-100 and blocked with 10% donkey serum. Primary antibody incubation was performed overnight at 4°C, and secondary antibody incubation for 1h at RT. A full list of primary antibodies is provided in Supplemental Table 1. Secondary antibodies were conjugated with Alexa Fluor 488, 568 and 647. Nuclei were counterstained with Hoechst 33258. Imaging was performed on a Zeiss LSM800 confocal microscope at the Biomedicum Imaging Core Facility, Karolinska Institutet.

**RNAscope on mouse motor neurons**

RNAscope (Wang et al., 2012) was used to verify the expression of Cox6a1 and Ybx1 based on the sequencing data. In brief, neural progenitors or motor neuron cultures, grown on glass coverslips or in microfluidic devices, were fixed with fresh PFA (4% in PBS) for 30min at 4°C. The RNAscope multiplex fluorescent kit v1 was used (Cat. No. 320850) according to the manufacturer's recommendations. To evaluate the procedure in neural progenitor and motor neurons cultures we tested the triple negative (Cat. 320871) and positive control probes (Polr2a-C1, Ppib-C2 and Ubc-C3) provided with the fluorescent kit in addition to a probe targeting poly-A tails of mRNAs (Cat. 318631-C2, 1 to 100 dilution). To easily visualize motor neurons, we combined the RNAscope procedure with immunofluorescence. After the hybridization with the last probe (Amp 4-FL) samples were washed and stained with an anti-GFP antibody.

Cells in microfluidic devices were stained with Cox6a1 (Cat. 519781-C2), Ybx1 (548711-C1) or Nrp1 (471621-C1) probes and the anti-GFP antibody. Representative images were taken with a Zeiss LSM800 confocal microscope at the Biomedicum Imaging Core Facility, Karolinska Institutet.

**Motor axon quantification**

The Fiji distribution of ImageJ (Schindelin et al., 2012) was used to analyze the areas of axonal GFP and MAPT (Tau) immunofluorescence. A ratio based on the respective areas was calculated and used for quantification. A total of five images, from three microfluidic devices, were quantified.

**Extended cDNA library preparation**

To prevent RNA degradation all procedures were carried out as rapidly as possible, and work surfaces and equipment were cleaned with RNaseZAP (AM9788M, Thermofisher) or DNAoff (9036, Takara). For all experiments, reagents were of molecular biology/PCR grade if available. Only tubes that were certified nuclease-free were used. Cell preparations harvested from the microfluidic devices were quickly thawed, vortexed and spun down. The volumes of axonal samples were always measured and 1µl of RNAse inhibitor was added before reducing the volumes from approximately 75µl to 12-15µl using a concentrator (5301, Eppendorf) set to aqueous solution mode at room temperature. For reverse transcription (RT) of axonal samples, 10µl of the lysates were used after concentration and the volumes of the reactions scaled-up accordingly (2X) to a final volume of approximately 20µl. Only 5µl of the lysates from somatodendritic preparations were directly used for the RT reactions, which were carried out in a final volume of 10 µl. Further library preparation of cell culture samples as well as human motor neurons for Illumina sequencing was carried out using the Smart-Seq2 protocol (Picelli et al., 2013) with some previously described modifications (Nichterwitz et al., 2016).

**Extended RNA-seq data processing and analysis**

Samples were sequenced on the Illumina HiSeq2000 or HiSeq2500 platforms, generating reads of 43 and 51bp, respectively. For mouse cell culture samples, reads were mapped to the mm10 mouse reference genome and the human SOD1 gene locus (hg38/GRCh38) with HISAT version 2.0.3 (Kim et al., 2015), using publicly available infrastructure from the main Galaxy server, available at *www.usegalaxy.org*. Aligned reads were then extracted and assigned using the GenomicAlignments package (version 1.8.4, (Lawrence et al., 2013)) in R, with the function

summarizeOverlaps, mode set to ‘union’. Normalized RPKM values were calculated using gene exon lengths from mm10 Ensembl version GRCm38.87 for mouse. For human samples, the hg38 reference genome was used for alignment. Quality control was performed on all samples and the exclusion criteria were: < 300,000 (axons) or < 500,000 (somas) uniquely mapped reads, Spearman correlation to other samples < 0.4 or < 2,500 genes expressed at RPKM > 0.1. If one or more of these criteria were not met, samples were excluded. To ensure that axonal samples to be used for analysis were not cross-contaminated with cell somas, during either the cell seeding process or the lysis, we conducted Spearman correlation, unsupervised hierarchical clustering and principal component analysis of all samples that passed the quality control. Axonal samples that clustered together with soma samples in these analyses were removed from further analyses. These samples typically displayed numbers of detected genes in the range for soma samples rather than other axonal samples.

Differential gene expression analysis was performed in R using DESeq2 version 1.12.4 (Love et al., 2014). Only genes with expression in at least four samples (irrespective of sample type) were considered for analysis. P-values were adjusted for multiple testing using the default Benjamini & Hochberg correction with FDR set to 10%, after which an adjusted p-value of < 0.05 was considered significant. All heatmaps were generated in R using *euclidean distance* as clustering method where applicable. Gene Ontology analyses were conducted using PantherDB (Mi et al., 2017) with the full *Mus musculus* background gene list. Gene set enrichment analyses (GSEA) were performed in R using the fgsea-package (version 1.2.1, available on: https://github.com/ctlab/fgsea). Gene sets were obtained from the Broad Institute Molecular Signatures Database (MSigDB v6.1, available on: https://software.broadinstitute.org/gsea/msigdb/). The used gene sets for testing were the hallmark (H), curated (C2) and GO-term-derived (C5) gene sets, as well as two custom gene sets for nuclear- and mitochondrial-encoded subunits for the respiratory electron chain. For comparison of mouse with human RNA-seq data, the Ensembl BioMart database was used to scan for orthologs between species. Only genes with known orthologs were used in the comparison to avoid inflating the proportion of non-overlapping genes.

**Use of published datasets**

All data used in cross-comparisons of datasets were obtained from the NCBI Gene Expression Omnibus except data pertaining to the study by Rotem et al. (2017), as no GEO submission was available. Here, a table of raw counts from a supplemental table of the article was used. The GEO accession numbers and their respective study

references for the axonal transcriptomics studies are as follows: GEO: GSE51572 (Minis et al., 2014), GEO: GSE66230 (Briese et al., 2016), GEO: GSE59506 (Saal et al., 2014). Data from single mESC-derived motor neurons was obtained from GEO: GSE76514 (Nichterwitz et al., 2016). Data from purified astrocytes, microglia and OPCs was obtained from GEO: GSE52564 (Zhang et al., 2014). In all cases for RNA-seq, data was remapped and RPKM values calculated using the pipeline described above, to avoid differential mapping bias. As this was not possible for the data from Rotem et al. the counts table provided was used.

## References

Briese, M., Saal, L., Appenzeller, S., Moradi, M., Baluapuri, A., and Sendtner, M. (2016). Whole transcriptome profiling reveals the RNA content of motor axons. Nucleic Acids Res *44*, e33.

Di Giorgio, F.P., Boulting, G.L., Bobrowicz, S., and Eggan, K.C. (2008). Human embryonic stem cell-derived motor neurons are sensitive to the toxic effect of glial cells carrying an ALS-causing mutation. Cell stem cell *3*, 637-648.

Guo, W., Naujock, M., Fumagalli, L., Vandoorne, T., Baatsen, P., Boon, R., Ordovas, L., Patel, A., Welters, M., Vanwelden, T*., et al.* (2017). HDAC6 inhibition reverses axonal transport defects in motor neurons derived from FUS-ALS patients. Nature communications *8*, 861.

Kim, D., Langmead, B., and Salzberg, S.L. (2015). HISAT: a fast spliced aligner with low memory requirements. Nature methods *12*, 357-360.

Kiskinis, E., Sandoe, J., Williams, L.A., Boulting, G.L., Moccia, R., Wainger, B.J., Han, S., Peng, T., Thams, S., Mikkilineni, S*., et al.* (2014). Pathways disrupted in human ALS motor neurons identified through genetic correction of mutant SOD1. Cell stem cell *14*, 781-795.

Lawrence, M., Huber, W., Pages, H., Aboyoun, P., Carlson, M., Gentleman, R., Morgan, M.T., and Carey, V.J. (2013). Software for computing and annotating genomic ranges. PLoS Comput Biol *9*, e1003118.

Love, M.I., Huber, W., and Anders, S. (2014). Moderated estimation of fold change and dispersion for RNA-seq data with DESeq2. Genome Biol *15*, 550.

Mi, H., Huang, X., Muruganujan, A., Tang, H., Mills, C., Kang, D., and Thomas, P.D. (2017). PANTHER version 11: expanded annotation data from Gene Ontology and Reactome pathways, and data analysis tool enhancements. Nucleic Acids Res *45*, D183-d189.

Minis, A., Dahary, D., Manor, O., Leshkowitz, D., Pilpel, Y., and Yaron, A. (2014). Subcellular transcriptomics-dissection of the mRNA composition in the axonal compartment of sensory neurons. Dev Neurobiol *74*, 365-381.

Nichterwitz, S., Chen, G., Aguila Benitez, J., Yilmaz, M., Storvall, H., Cao, M., Sandberg, R., Deng, Q., and Hedlund, E. (2016). Laser capture microscopy coupled

with Smart-seq2 for precise spatial transcriptomic profiling. Nature communications *7*, 12139.

Picelli, S., Bjorklund, A.K., Faridani, O.R., Sagasser, S., Winberg, G., and Sandberg, R. (2013). Smart-seq2 for sensitive full-length transcriptome profiling in single cells. Nature methods *10*, 1096-1098.

Rotem, N., Magen, I., Ionescu, A., Gershoni-Emek, N., Altman, T., Costa, C.J., Gradus, T., Pasmanik-Chor, M., Willis, D.E., Ben-Dov, I.Z.*, et al.* (2017). ALS Along the Axons - Expression of Coding and Noncoding RNA Differs in Axons of ALS models. Sci Rep *7*, 44500.

Saal, L., Briese, M., Kneitz, S., Glinka, M., and Sendtner, M. (2014). Subcellular transcriptome alterations in a cell culture model of spinal muscular atrophy point to widespread defects in axonal growth and presynaptic differentiation. RNA *20*, 1789-1802.

Schindelin, J., Arganda-Carreras, I., Frise, E., Kaynig, V., Longair, M., Pietzsch, T., Preibisch, S., Rueden, C., Saalfeld, S., Schmid, B.*, et al.* (2012). Fiji: an open-source platform for biological-image analysis. Nature methods *9*, 676-682.

Thams, S., Lowry, E.J., Larraufie, M-H., Spiller, K.J., Li, H., Williams, D.J., Hoang, P., Jiang, E., Williams, L.A., Sandoe, J., *et al.* (2018). A stem cell-based screening platform identifies compounds that desensitize motor neurons to endoplasmatic reticulum stress. Molecular Therapy. doi: https://doi.org/10.1016/j.ymthe.2018.10.010

Wang, F., Flanagan, J., Su, N., Wang, L.C., Bui, S., Nielson, A., Wu, X., Vo, H.T., Ma, X.J., and Luo, Y. (2012). RNAscope: a novel in situ RNA analysis platform for formalin-fixed, paraffin-embedded tissues. The Journal of molecular diagnostics : JMD *14*, 22-29.

Zhang, Y., Chen, K., Sloan, S.A., Bennett, M.L., Scholze, A.R., O'Keeffe, S., Phatnani, H.P., Guarnieri, P., Caneda, C., Ruderisch, N.*, et al.* (2014). An RNA-sequencing transcriptome and splicing database of glia, neurons, and vascular cells of the cerebral cortex. J Neurosci *34*, 11929-11947.
